# The association between low birth weight and dental caries among 11-to-13-year-old school age children in Ningbo, China

**DOI:** 10.1186/s12887-021-02968-7

**Published:** 2021-11-04

**Authors:** Xiaoyan Weng, Yiting Lou, Ran Tao, Yongzheng Li, Danna Cao, Mengfei Yu, Binbin Ying, Huiming Wang

**Affiliations:** 1 The Department of Stomatology, The Affiliated Ningbo Hospital of Zhejiang University, Ningbo, 315000 China; 2 The Affiliated Hospital of Stomatology, School of Stomatology, Zhejiang University School of Medicine, and Key Laboratory of Oral Biomedical Research of Zhejiang Province, Zhejiang, 310006 Hangzhou China; 3 The Department of Human Anatomy, Laboratory of Clinical Applied Anatomy, School of Basic Medical Science, Fujian Medical University, Fuzhou, 350000 China

**Keywords:** Low birth weight, Dental caries, Permanent teeth, Children, China

## Abstract

**Background:**

The association between low birth weight (LBW) and dental caries is currently unclear. The aim of this study was to investigate the association of LBW with dental caries in permanent teeth in children of Ningbo city.

**Methods:**

A total of 1975 children aged 11-to-13 years in Ningbo, China were enrolled in this cross-sectional study. LBW was defined as a birthweight< 2500 g. Ten dentists assessed the status of dental caries in permanent teeth in line with the World Health Organization (WHO) criteria and guidelines. Decayed, missing or filled teeth were considered to have dental caries. Parental questionnaires were used to collect child information. Non-conditional logistic regression analysis was used to estimate odds ratios (ORs) and the corresponding 95% confidence intervals (CIs).

**Results:**

Dental caries in permanent teeth was found in 610 children (30.9%), with a mean DMFS of 2.09 (SD = 1.2). The adjusted ORs for dental caries in permanent teeth was 1.46 (95% CI 1.00, 2.13) for LBW.

**Conclusions:**

LBW was not associated with dental caries in permanent teeth in the study population.

## Introduction

Low birth weight (LBW) risk factors, such as maternal diabetes, hypertension, obesity, and older age, have been on the rise, making LBW a major public health issue in China [1]. A 2013 epidemiological survey showed that the incidence of LBW (common in preterm babies) in China was 6.1% (< 2500 g) [2]. Advances in technology have decreased the mortality rate due to LBW. However, complications, such as respiratory disorders and oral problems, have been reported in patients with LBW [3, 4]. Previous studies found that LBW and preterm children result in poor dental health and hygiene [4].

The incidence of dental caries is influenced by genetic, socioeconomic and early life factors [5–8]. Prematurity and LBW have been found to only affect the enamel structure of the primary teeth since permanent teeth mineralize only after birth. In recent years, some studies have shown that birth outcomes affect the ameloblasts during the secretory or maturation phase of tooth development, causing both hypoplastic and hypomineralized enamel [9–12]. The defects affect the permanent incisors and first molars during permanent dentition. To date, the association of LBW with dental caries is unclear [4, 13–16]. Furthermore, as far as we know, no study has investigated this in China.

Therefore, we investigated the association of LBW with dental caries in permanent teeth of Chinese children.

## Methods

### Design

The following formula was used to calculate the required sample size:

$$n= deff\frac{\mu^2p\left(1-p\right)}{\varepsilon^2}$$

Where *n* is the sample size, *deff* is the design effect set as 1.5, *p* is the prevalence of dental caries, *μ* is the level of confidence, and *ε* is the margin of error. The prevalence of dental caries is 28.9% according to the Third National Oral Survey of China. The non-response rate is sets as 20%. Based on this estimation, the final sample size was 1813.

The study was conducted on 11-to-13-year-old children from 10 regions involved in the pit and fissure closure treatment in 2020, public welfare activity for school-age children in Ningbo city. Pit and fissure closure treatment prevents dental caries, mainly in the pits and fissures of occlusal tooth surfaces. The participants were recruited from each region, using a stratified sampling method. LBW was defined as having a birth weight < 2500 g, as proposed by Mikolajczyk [17]. The children were diagnosed with caries in permanent teeth if the permanent incisors or first molars were decayed, missing, or filled [18]. Written informed consent was obtained from all children and parents. The study was conducted in line with Studies in Epidemiology (STROBE) guidelines for reporting observational studies [19].

### Oral examination

Ten dentists from The Affiliated Ningbo Hospital of Zhejiang University performed the clinical examinations on the children. A visual examination using optimal lighting, a mouth mirror, and a probe in a supine position (no X-rays) was carried out to assess dental caries. Teeth were dried before the inspection to guarantee accurate results. Dental caries was measured using decayed, missing and filled surfaces (DMFS) following the WHO criteria [20].

### Quality control

An experienced dentist (B.Y) trained the ten examiners to equip them with theoretical and clinical knowledge before examination. Each examiner was calibrated with the standard examiner (B.Y) and other examiners by assessing 50 children on each of 2 occasions, 1 month apart. These 50 children were enrolled in this study and were randomly selected. Additionally, 5% of the samples were randomly reexamined to monitor inter-examiner and intra-examiner reproducibility.

### Questionnaire survey

A structured questionnaire was used to collect information such as age, gender, birth weight, dental health behavior (the frequency of sugary food intake, toothbrushing frequency, age at first brushing, regular dental check-ups, fluoride use), the maternal exposure to tobacco smoke during pregnancy, parental educational level.

Fluoride use was classified as positive when children used fluoride agents at home, such as toothpaste and gel, or topical fluoride products at hospital. Birth weight was filled according to the Maternal and Child Health Handbook (booklet for pregnancy, delivery and postnatal/child health) issued by the Zhejiang Province Department of Health.

### Statistical analysis

Chi-square or Fisher’s exact tests were used to compare subjects’ characteristics. Gender, parental educational level, maternal exposure to tobacco smoke during pregnancy, the frequency of sugary food intake, toothbrushing frequency, age at first brushing, fluoride use, regular dental check-ups were considered confounding factors based on existing literature. Non-conditional logistic regression analysis was used to estimate crude and adjusted odds ratios (ORs) with 95% confidence intervals (95%CIs) to determine the association of LBW with dental caries. DMFS was the dependent variable for the non-conditional logistic regression analysis. “DMFS = 0″ was denoted as “0″, and “DMFS> 0″ was denoted as “1″. The SAS software Version 9.4 (SAS Institute, Cary, North Carolina, USA) was used for all statistical analysis.

## Results

A total of 8702 11-to-13-year-old school age children received pit and fissure closure and their parents were provided with a questionnaire. Two thousand one questionnaires subsequently recruited from 10 regions, using a stratified sampling method (23.0%). Missing answers and illogical data were resolved by contacting parents via a telephone interview. A total of 1975 children (22.7%) with complete information were enrolled. The Kappa values used to assess inter-examiner and intra-examiner reproducibility ranged from 0.81 to 0.96.

The mean age of the 1975 children was 11.3 (SD = 0.5). Among them were 1013 (51.2%) males and 962 (48.8%) females. A total of 610 children (30.9%) had dental caries in permanent teeth. Specifically, the prevalence of dental caries in LBW children and normal birth weight (NBW) children was 38.3 and 30.3%, respectively. The mean DMFS of all subjects was 0.65 (SD = 1.2), and the mean DMFS of LBW children and NBW children was 0.84 (SD = 1.3) and 0.63 (SD = 1.2), respectively.

The sample and the population distributions in the 10 Ningbo Province regions are shown in Table 1. The characteristics of the study population are prescribed in Table 2. The mean birth weight of the entire study sample was 3, 332.1 g, and the prevalence of LBW was 6.5%. About 70% of the subjects consumed sugary foods less than once a day, and more than 40% were over 3 years old before starting to brush. About 74% of children brushed teeth ≥2 times daily, and 70% received dental examination at least once a year. Approximately 54% of the children used fluoride agents. Of note, most dietary habits and lifestyles were similar between LBW children and NBW children.

**Table 1:** Distribution of the sample and the population in the 10 regions of Ningbo

	Numbers of sample		Number of Population by Region	
	(n)	%	(n)	%
**Beilun**	609	7.0	429,700	7.1
**Cixi**	1638	18,8	1059,600	17.4
**Fenghua**	673	7.7	480,600	7.9
**Haishu**	951	10.9	633,500	10.4
**Jiangbei**	378	4.3	262,800	4.3
**Ninghai**	877	10.1	633,900	10.4
**Xiangshan**	747	8.6	560,400	9.2
**Yingzhou**	1376	15.8	931,000	15.3
**Yuyao**	1071	12.3	835,900	13.7
**Zhenhai**	382	4.4	257,600	4.3

**Table 2:** Distribution of selected characteristic in 1, 975 children

		Low birth weight		Normal birth weight		Total		
	Category	(n)	%	(n)	%	(n)	%	*P*
**Variable**								
**Total**		128	6.5	1847	93.5	1975	100	
**Sex**	Female	61	47.7	901	48.8	962	48.1	.805
	Male	67	52.3	946	51.2	1013	51.3	
**Paternal educational level (years)**	< 9	47	36.7	745	40.3	792	40.1	.407
	9-12	45	35.2	546	29.6	591	29.9	
	> 12	36	28.1	556	30.1	592	30.0	
**maternal exposure to tobacco smoke during pregnancy**	Active smoking	0	0.0	16	0.8	16	0.8	<.001
	Passive smoking	62	48.4	141	7.6	203	10.3	
	No smoking	66	51.6	1690	91.5	1756	88.9	
**The frequency of sugary food intake (times/day)**	< 1	90	70.3	1286	69.6	1376	69.7	.782
	1-2	28	21.9	383	20.7	411	20.8	
	> 2	10	7.8	178	9.6	188	9.5	
**Age at first brushing (years old)**	2	29	22.7	480	26.0	509	25.8	.065
	3	35	27.3	635	34.4	670	33.9	
	> 3	64	50.0	732	39.6	796	40.3	
**Toothbrushing frequency (times/day)**	0	1	1.0	18	1.0	19	1.0	.398
	1	38	21.9	450	24.4	488	24.7	
	> 2	89	60.5	1379	74.7	1468	74.3	
**Dental check-ups (times/year)**	0	38	30.0	476	25.8	514	26.0	.583
	1	57	44.5	842	45.6	899	45.5	
	2	33	25.8	529	28.6	562	28.5	
**Fluoride use**		53	41.4	1016	55.0	1069	54.1	.003

The association of LBW with dental caries in permanent teeth is shown in Table 3. There was no association between LBW and dental caries in permanent teeth after adjusting for confounding factors.

**Table 3:** ORs and 95% CIs for the association between dental caries in permanent teeth and LBW

		Prevalence	Unadjusted ORs [95%CI]	Adjusted ORs [95%CI]
**Low birth weight**	Yes	49/128 (38.3%)	1.43 [0.96,2.06]	1.46 [1.00,2.13]
	No	560/1847 (30.3%)	1.00	1.00

*ORs* odds ratios, *95% CI* 95% confidence interval

Adjusted for gender, paternal educational level, maternal exposure to tobacco smoke during pregnancy, the frequency of sugary food intake, age at starting to brush, toothbrushing frequency, dental check-up, use of fluoride

## Discussion

In this cross-sectional study, LBW was not associated with dental caries. This is consistent with findings in a retrospective cohort research in the USA, which found no increased risk of caries in very low birth weight (VLBW) group [3]. However, the study in USA did not distinguish posteruptive enamel loss in hypomineralized areas from caries in permanent molars and incisors. A previous retrospective cohort study conducted in Dubai found different results. It was found that enamel defects and dental caries in permanent teeth were significantly higher in the preterm group than in the full-term group in 5-to-10-year-old children [21]. Hypomineralization has been found to increases susceptibility to dental caries. The relationship between LBW and hypomineralization is currently unclear. A cross-sectional study involving 420 8 years old children in Thailand reported that LBW was not associated with molar incisor hypomineralization [22] while other similar studies found an association between the two [23, 24].

Several factors may explain these inconsistencies. For instance, some studies may have examined for dental caries at an age when dental caries had not developed, hence the association of LBW and dental caries was not detected [25, 26]. Therefore, the age of caries assessment should be investigated in future studies. Kay [27] reported that dental caries should be assessed at least 2 years after teeth eruption, to provide enough time for teeth decay. The inconsistencies may also be caused by different methodologies across studies. Nondifferential measurement error cannot be avoidable in a cross-sectional study. Furthermore, different confounding factors, which limits the feasibility of inter-study comparisons. Therefore, findings from previous studies are inconsistent and cannot be used to draw a clear conclusion applicable to all populations.

This study examined the relationship between LBW and dental caries in permanent teeth among children aged 11-13 years. Previous investigations focused on the influence of LBW on primary teeth [13, 21, 28]. The impacts of LBW on permanent teeth, especially permanent incisors and first molars, primarily originate from the secretory phase of the ameloblast. It begins in utero, and the maturation phase starts at birth. If inadequate mineralization or trauma occurs during these periods, the enamel defects appear, increasing the risk of caries [3, 29].

Several factors may explain the lack of association between LBW and dental caries in permanent teeth. LBW babies receive more attention and adequate nutritional intake from guardians than NBW infants [30]. Besides, their guardians are more proactive in implementing health and oral health instructions, consistent with findings by Gravina [31]. Specifically, Gravina [31] showed that premature children have lower rates of caries than term children because of appropriate oral health care and regular pediatric care. Furthermore, premature birth and LBW cause delayed teeth eruption, reducing caries information, thus minimizing the risk of dental caries [30, 32].

This study has several advantages. The participants were homogeneous in terms of age and geographic background. Detailed information, such as the frequency of sugary food intake, the frequency of brushing and starting age, regular dental check-ups were collected, making it easier to control for confounding factors. Birth weight was obtained from the hospital health booklet, suggesting that the data was accurate. Besides, the study included a large number of participants, which reduces contingency.

Nevertheless, there are some limitations worth mentioning. The outcomes may vary across different populations due to various cultural and social factors. The participants were from an economically developed area, and hence the present findings may not be applicable to other sites. Moreover, some limitations of a cross-sectional study such as selection bias and recall bias, could not be avoided. Furthermore, we did not assess the small for gestational age (SGA) that precisely evaluates intrauterine growth restriction [33].

## Conclusion

In this cross-sectional study, LBW was not associated with dental caries in permanent teeth in children from Ningbo, Zhejiang province. However, it is likely that such an association exists in other areas. Further prospective studies should be conducted to verify the relationship between the LBW and dental caries in permanent teeth.

## Data Availability

The data from this study is available with the corresponding authors and can be accessed on request.

## Abbreviations

LBW: Low Birth Weight
WHO: World Health Organization
ORs: Odds ratios
95% CIs: 95% Confidence Intervals
NBW: Normal Birth Weight
VLBW: Very Low Birth Weight
SGA: Small for Gestational Age
